# Prediction Model Risk-of-Bias Assessment Tool for coronary artery lesions in Kawasaki disease

**DOI:** 10.3389/fcvm.2022.1014067

**Published:** 2022-10-13

**Authors:** Hongbiao Huang, Jinfeng Dong, Shuhui Wang, Yueping Shen, Yiming Zheng, Jiaqi Jiang, Bihe Zeng, Xuan Li, Fang Yang, Shurong Ma, Ying He, Fan Lin, Chunqiang Chen, Qiaobin Chen, Haitao Lv

**Affiliations:** ^1^Department of Pediatrics, Fujian Provincial Hospital, Fujian Provincial Clinical College of Fujian Medical University, Fuzhou, Fujian, China; ^2^Department of Pediatrics, Institute of Pediatric Research, Children’s Hospital of Soochow University, Suzhou, Jiangsu, China; ^3^Department of Hematology, The First Affiliated Hospital of Fujian Medical University, Fuzhou, Fujian, China; ^4^Department of Epidemiology and Biostatistics, School of Public Health, Medical College of Soochow University, Suzhou, Jiangsu, China; ^5^Department of Pediatrics, Fuqing City Hospital Affiliated to Fujian Medical University, Fuqing, Fujian, China

**Keywords:** coronary artery lesions, Kawasaki disease, Prediction Model Risk-of-Bias Assessment Tool, prediction model, diagnosis

## Abstract

**Objective:**

To review and critically appraise articles on prediction models for coronary artery lesions (CALs) in Kawasaki disease included in PubMed, Embase, and Web of Science databases from January 1, 1980, to December 23, 2021.

**Materials and methods:**

Study screening, data extraction, and quality assessment were performed by two independent reviewers, with a statistics expert resolving discrepancies. Articles that developed or validated a prediction model for CALs in Kawasaki disease were included. The Critical Appraisal and Data Extraction for Systematic Reviews of Prediction Modeling Studies checklist was used to extract data from different articles, and Prediction Model Risk-of-Bias Assessment Tool (PROBAST) was used to assess the bias risk in different prediction models. We screened 19 studies from a pool of 881 articles.

**Results:**

The studies included 73–5,151 patients. In most studies, univariable logistic regression was used to develop prediction models. In two studies, external data were used to validate the developing model. The most commonly included predictors were C-reactive protein (CRP) level, male sex, and fever duration. All studies had a high bias risk, mostly because of small sample size, improper handling of missing data, and inappropriate descriptions of model performance and the evaluation model.

**Conclusion:**

The prediction models were suitable for the subjects included in the studies, but were poorly effective in other populations. The phenomenon may partly be due to the bias risk in prediction models. Future models should address these problems and PROBAST should be used to guide study design.

## What is already known on this topic

Almost all CAL prediction models performed well in the included population but were poorly effective in other populations.

This phenomenon is partly due to the differences in the genetic backgrounds of the populations.

## What this study adds

In addition to differences in genetic background, bias in model development may be one of the major reasons for the lack of efficacy of the models in different populations.

This is the first time PROBAST has been applied for the evaluation of Kawasaki disease.

PROBAST revealed a high risk of bias in all studies, mostly because of small sample size, improper handling of missing data, and inappropriate descriptions of model performance and the evaluation model.

## How this study might affect research, practice or policy

The risk of bias may be one of the reasons for the poor performance of the model in different populations.

Larger sample sizes, external data validation, better processing of missing values, and the use of appropriate model construction methods are the methods to reduce the bias of prediction models.

Future prediction models should pay more attention to these problems, and PROBAST can be used to guide study design.

## Introduction

Kawasaki disease (KD) is an acute, self-limiting form of vasculitis that affects children, particularly those aged < 5 years (1). Medium-sized arteries, particularly coronary arteries, are the most vulnerable vessels in this disease (2). The incidence of coronary artery lesions (CALs) in affected patients has decreased significantly because of intravenous immune globulin (IVIG) therapy; however, they still occur in 4–6% of patients (2). The incidence of CALs is also closely related to cardiovascular diseases in adulthood, especially coronary heart disease (3). Patients believed to be at high risk for the development of these lesions may benefit from more aggressive primary adjunctive therapy, particularly increased additional anti-inflammatory medications including corticosteroids and infliximab (4). As in the case of IVIG resistance prediction (4), some Japanese risk models were found to be poorly effective in predicting CAL development in a Western population (5). On the one hand, this phenomenon may be partly attributable to racial and other differences between the study populations (1); on the other hand, several studies have reported that the quality of previous research on prediction models was poor (6). For these reasons, although a large number of articles on prediction models are published every year, only a very number them have been used (7). Therefore, a systematic review of these CAL prediction models and an effective model evaluation tool are very important.

In this systematic review, we aimed to minimize subjectivity and bias inherent in related previous research (8, 9). The Prediction Model Risk-of-Bias Assessment Tool (PROBAST) is useful for assessing systematic reviews of prediction model studies and critically appraising (primary) prediction model studies (10). PROBAST contains four domains with 20 signaling questions to assess the risk of bias for the prediction of CALs in KD. In this work, PROBAST was used to evaluate the reported CAL prediction models so as to identify a more effective prediction model for application in clinical treatment. This work is the very first instance of the application of PROBAST in KD research. To date, an assessment of the risk of bias (ROB) in prediction models for KD has not been reported. Therefore, as stated previously, this work was aimed at comprehensively evaluating the performance and bias of CAL prediction models in KD.

## Materials and methods

### Search strategy

English articles included in the PubMed, Embase, and Web of Science databases from January 1, 1980, to December 23, 2021, were searched using the following keywords: (“Kawasaki disease” OR “mucocutaneous lymph node syndrome”) AND (“coronary artery dilatation” OR “coronary artery aneurysms” OR “coronary artery lesions”) AND (“predict” OR “score” OR “nomogram”). As described previously (8), the list was validated to examine whether it was fit for purpose by comparing it to relevant hits from the said databases when combining KD (KD, mucocutaneous lymph node syndrome) and coronary artery lesion (“coronary artery dilatation” OR “coronary artery aneurysms” OR “coronary artery lesions”) search terms with methodological search terms (“predict” OR “score” OR “nomogram”). The aims of these studies were to determine the risk factors for coronary artery injury and to improve the prognosis of KD.

### Inclusion and exclusion criteria

All of the studies that focused on the prediction of CALs in KD were included. The inclusion criteria were as follows: (1) predictive models established for populations with KD, (2) a diagnosis of KD and CALs established per diagnostic criteria, and (3) inclusion of at least two predictors in the prediction model, because PROBAST was designed to assess multivariable prediction models for diagnoses and doctors usually make predictions by integrating several characteristics and symptoms (6, 10). Studies that met the following criteria were excluded: (1) studies that did not include information on clinical outcomes; (2) studies that only included a single predictor; (3) studies that did not include animal research; (4) non-inclusion of reviews, case reports, and meta-analysis; (5) studies that investigated the same object, and (6) studies in which the predictors did not include genes, because there are large differences in genetic susceptibility to KD and genetic testing is not routinely performed in the treatment of KD (4).

### Study selection and data extraction

Three researchers (HH, DJ, and WS) screened studies to determine if they developed or validated a multivariable model or scoring system to predict any CAL-related outcome, respectively. Any differences were resolved through discussion or by a statistics expert (SY). Then, the Critical Appraisal and Data Extraction for Systematic Reviews of Prediction Modeling Studies (CHARMS) checklist was used for data extraction (11). The PROBAST form was used to evaluate the bias of the prediction models (10). Some methods and principles derived from Preferred Reporting Items for Systematic Reviews and Meta-analyses (PRISMA) (12) and Transparent Reporting of a Multivariable Prediction Model for Individual Prognosis or Diagnosis (TRIPOD) (6) were used. Information was extracted from each prediction model, and predictive performance was evaluated by the type of validation, discrimination, and calibration (8).

### Patient and public involvement statement

Patients and/or the public were not involved in the design, or conduct, or reporting, or dissemination plans of this research.

## Results

**FIGURE 1:**
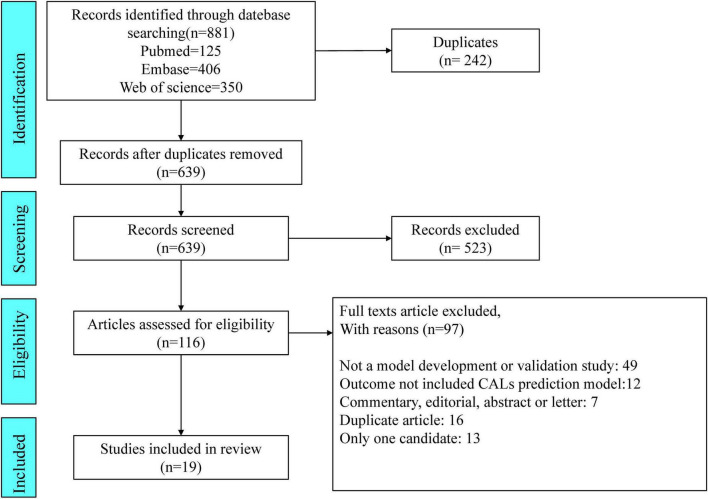
Preferred Reporting Items for Systematic Reviews and Meta-analyses (PRISMA) flowchart of the study inclusions and exclusions.

We identified 881 articles through our search of PubMed (*n* = 125), Embase (*n* = 406), and Web of Science (*n* = 350). Of the 881 articles, 19 pertaining to studies describing CAL prediction models met the inclusion criteria and were selected for data extraction and critical appraisal (5, 13–30). In Figure 1, the flowchart, in line with PRISMA, shows our retrieval process.

### Study characteristics

**FIGURE 2:**
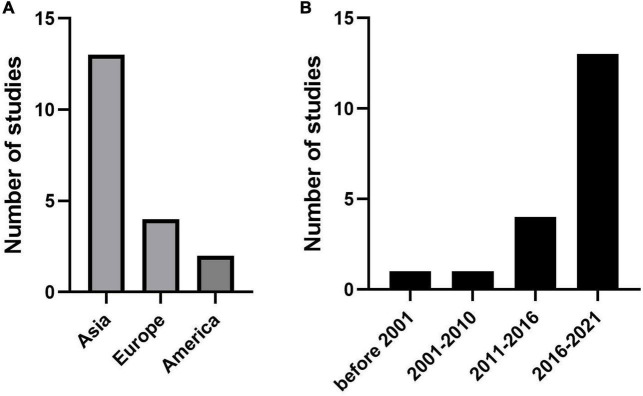
Publications trends for the coronary artery lesion (CAL) prediction model. **(A)** The number of studies in different regions. **(B)** The number of studies in different years.

Thirteen studies used data on patients with KD from different regions of Asia, such as China (20, 27, 29), Taiwan (16, 22, 24), Japan (13, 21, 25), Korea (14, 26, 30), and Thailand (18). Four studies were published in Europe, including Poland (15), Spain (19), Turkey (23) and France (28). The remaining two articles were for studies from the USA (5, 17) (Figure 2A). Almost all articles had been published in the last 10 years (from 2012 to 2021), except two articles (13) (26) published in 1986 and 2007, respectively (Figure 2B). The duration of follow-up was significantly different, extending from immediately after treatment (21) to 3 months after treatment (29). The number of patients included ranged from 73 (15) to 5,151 (30). All study designs were based on retrospective cohorts. The median age, sex proportion, and other details are shown in Supplementary Table 1.

### Methods of development and validation

Most studies included used logistic regression. One study used discrimination analysis (13), while two (17, 23) used multivariable analysis. Only one study used machine learning methods. The machine learning methods included the mean structure equation model and neural networks. All but two studies (5, 21) did not use external data to validate the developed model.

### Predictors in the final model

Different prediction models provided different predictors. The most frequently included predictors were C-reactive protein (CRP) level, male sex, and fever duration. Seven articles reported that these factors indicated a high risk of CALs. Platelet count, age, and IVIG resistance were considered as risk factors for CALs in six articles each. The other predictors that deserved attention included the baseline *Z* score and the day of the beginning of treatment. Those two predictors were screened out by more than three articles each.

### Definition of coronary artery lesions and model performance

The diagnosis of CALs showed changes over the long period of the literature included in the study, resulting in different diagnostic criteria for CALs in different articles. However, these differences in CAL diagnoses were based on the relevant diagnostic guidelines at that time. Because the *Z* score was clinically used for a short time, less than half of the articles (7/19) defined CALs by *Z* score (5, 14, 16, 17, 21, 24, 25). Four of these articles were published in the last 3 years. The remaining articles used diameter to assess the occurrence of CALs. Almost half of these article outcomes (9/19) to assess the performance of CALs models were sensitivity, specificity, and AUC (area under the curve of a receiver operating characteristic curve). The ranges were as follows: sensitivity, 25.0% (30) to 87.5% (13); specificity, 68.2% (20) to 99% (21); and AUC, 0.52 (30) to 0.86 (24).

### Risk of bias

**FIGURE 3:**
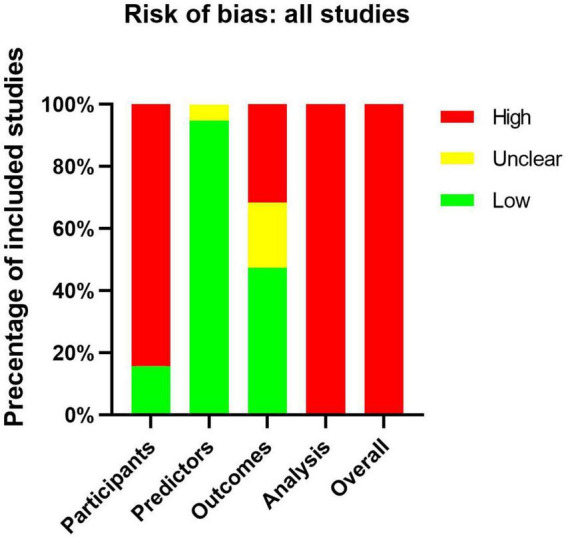
Risk-of-bias assessment of the included studies by using the Prediction Model Risk of Bias Assessment Tool (PROBAST).

All articles assessed by PROBAST tools be considered as high ROB (Supplementary Table 2). This result suggests that the poor applicability of the CAL prediction model may be related to the bias of the model itself. Sixteen of the 19 articles had a high ROB for the participants’ domain, because these articles used existing data which were considered cause ROB easily (10). Only three articles (19, 25, 30) used registration data which were recommended by PROBAST. Eighteen of the 19 articles had a low ROB for the predictor domain. The reason for unclear was that one of the predictors they included was IVIG resistance, but they evaluated the CALs from onset to 1 month. IVIG resistance may be an unavailable predictor when the onset of disease course because we did not know whether the IVIG resistance would happen. About the outcome domain, nine of the 19 articles had a low ROB. Four of the 19 articles were assessed as unclear because they did not explain the diagnostic criteria of KD. We should make clear the correct diagnosis of KD before we evaluate the classification of CALs. Six of the 19 articles used the *Z* score or diameter of the coronary artery as predictors, predictors were very similar to the outcome definition should be considered a high ROB (Figure 3).

All studies showed a high ROB for the analysis domain (Table 1), which can be attributed to many reasons. In our study, the small number of cases in training set, dichotomization of continuous predictors, lack of external validation, the use of univariable analysis to select predictors, and direct deletion of missing data were the most commonly identified deficiencies. Such serious deficiencies can yield prediction models that are unsuitable for clinical application (6). According to the event per variable (EPV) principle to evaluate the effective sample size, eight of the 19 articles had appropriate EPVs of at least 20. Only five articles did not switch the continuous predictors into classified data. None of those articles dealt with missing data with an appropriate method like multiple imputations. Three articles avoided the use of univariable analysis: two (17, 30) used multivariable logistic regression directly and one (21) used neural network analyses. Moreover, none of these articles used calibration plots or tables to calibrate the prediction model, and only two articles (5, 17) evaluated the calibration by Hosmer–Lemeshow test. Six articles evaluate the discrimination by meaning the C-index or AUC. Nine articles evaluated the classification on the basis of sensitivity and specificity. Only two articles (5, 21) used external data to validate the model performance.

**TABLE 1:** Risk of bias assessment (using PROBAST) based on four domains across 27 studies that created CALs prediction models for Kawasaki disease.

Authors	Risk of bias			
	
	Participants	Predictors	Outcome	Analysis
Nakano et al. (13)	High	Low	Unclear	High
Kim et al. (26)	High	Low	Low	High
Ruan et al. (27)	High	Unclear	Low	High
Lega et al. (28)	High	Low	Low	High
Xu et al. (29)	High	Low	Unclear	High
Kim et al. (14)	High	Low	Unclear	High
Berdej-Szczot et al. (15)	High	Low	Low	High
Liu et al. (16)	High	Low	High	High
Son et al. (17)	High	Low	High	High
Kim et al. (30)	Low	Low	Low	High
Chantasiriwan et al. (18)	High	Low	High	High
Hua et al. (20)	High	Low	Low	High
Fernandez-Cooke et al. (19)	Low	Low	Low	High
Son et al. (5)	High	Low	High	High
Turkucar et al. (23)	High	Low	Low	High
Chang et al. (22)	High	Low	Low	High
Azuma et al. (21)	High	Low	Unclear	High
Huang et al. (24)	High	Low	High	High
Lio et al. (25)	Low	Low	High	High

## Discussion

In this study of prediction models related to CALs in KD, we critically appraised 19 studies using PROBAST. Although these prediction models may have been suitable for the patients included in the respective studies, they were all appraised to have high ROB owing to different reasons, including small sample sizes, improper handling of missing data, and inappropriate description of model performance and evaluation model. Therefore, we have discussed the common causes of biases in prediction model, with the aim of providing references and suggestions for building a more practical CAL prediction models in the future.

### Sample size

A major reason for the poor performance of the CAL prediction models in different populations was that the sample size was insufficient, and the EPV values were often less than 10, which made the model prone to overfitting. An overfitting model is too closely tailored to the training data that yields inaccurate predictions in other populations (11). Eight of the 19 articles had adequate EPVs, which may indicate us more people should be included to develop a more valuable prediction model.

### Candidate predictors

Differences in the definitions and measurement methods of candidate predictors may also lead to the poor prediction performance in different populations (11). In our study, the definitions of CALs were different, and ranged from evaluation of diameters to measurement of *Z* scores. Moreover, the measurement methods involved different evaluation times and intervals of cardiac ultrasound after treatment with IVIG, different pediatric echocardiographers and different cardiac color Doppler ultrasound machines, which may also lead to poor prediction efficiency in different populations.

### Missing data

Many studies simply deleted participants or candidate predictors with a number of missing data, which yielded a non-random subset of the original study sample, and produced inefficient prediction performance (31). Multiple imputation is strongly recommended as a preferred method for handling missing data (11, 31). Unfortunately, none of the articles assessed in our study used this method. One article used expectation maximum estimated statistics (27). The inappropriate deletion of missing data was another reason for the poor performance of the prediction model.

### Model development

Because the CHARMS checklist reported that model development should not be based on univariable testing, which causes a severe risk of predictor selection bias (11), we designed the exclusion criteria by including only one predictor. However, the approaches for handling continuous predictors were still unsatisfactory. Many statements recommended keeping continuous variables continuous instead of converting them into categorical variables, which reduces predictive ability (10, 32). Only one article used a machine learning methods for analysis (21). Greater application of machine learning methods and less univariate analysis may be more conducive to the development of prediction models.

### Model performance

Calibration and discrimination are often used to measure prediction model performance, and classical indicators such as sensitivity and specificity are used to evaluate the model classification abilities (10). Eight of the 19 articles used sensitivity and specificity to evaluate model performance, but different thresholds produced different values for sensitivity and specificity. The choice of the threshold should be based on the general principle rather than the data itself. Instead, the studies determined the threshold on the basis of the data itself, which may be another reason for the over-optimistic estimates of model performance. None of the articles used a calibration plot to display the model’s calibration. Two articles used only the Hosmer–Lemeshow test to calibrate the prediction model even though this test can be considered to yield poor calibration (10). Discrimination is usually assessed using C-index or AUC, since these two indicators have the same sense. Six articles used this indicator to evaluate the model performance. Due to these situations, more appropriate methods to evaluate the performance of the model, such as the calibration plot and C-index, should be included in future studies on prediction model construction.

### Model evaluation

Analyses using an independent validation dataset are essential to avoid developing an overestimating model. The validation process should consist of both internal and external validation. Internal validation usually includes bootstrapping and cross-validation, while external validation differs in time (temporal validation) or location (geographical validation) from the data resource (10). Bootstrapping has been shown to be more effective in small datasets (10). In comparison with temporal validation, geographical validation has been more recommended in external validation (11). Two articles used external validation, and employed geographical validation to assess their models (5, 21). Thus, further development and verification of CAL prediction models using both internal and external validation is essential.

## Conclusion

Although the reported prediction models for CALs in KD were appropriate in their respective studies, they did not perform well in other populations. In addition to the differences in genetic backgrounds, the authors’ overly optimistic evaluation models may be another reason for these discrepancies. In assessments performed using PROBAST tools, all of these models showed high ROB, mainly because of the small sample size, improper handling of missing data, inappropriate description of model performance, and inappropriate evaluation model cause the performance of model is likely to be misleading and optimistic. Future prediction models should address these problems and use PROBAST to guide the study design.

## Limitations

Our study had several limitations. First, most articles (14/19) had been published before the PROBAST tool was published in 2019, so the authors of those articles could not have used this tool to assess their studies, potentially increasing the proportion of studies classified as showing a high ROB. Second, KD has obvious genetic background differences; thus, it is impossible to build a perfect model only by correcting the model bias. Third, our research included articles published in English, it may cause bias. Last, ROB judgment is subjective sometimes. Different raters may draw different conclusions, when we close the decision by a statistics expert and ruled out other raters’ conclusions may lead to another type of bias. And more reasonable methods need to be considered.

## Data availability statement

The original contributions presented in this study are included in the article/Supplementary material, further inquiries can be directed to the corresponding authors.

## Author contributions

HH, JD, and SW conceived the idea of the study and screened the studies. YS supervised the analysis. YZ, JJ, BZ, XL, FY, SM, YH, FL, and CC performed data collection. HH drafted the manuscript. QC and HL critically reviewed and revised this manuscript. All authors contributed to the article and approved the submitted version.

## Abbreviations

CALs: coronary artery lesions
PROBAST: Prediction Model Risk-of-Bias Assessment Tool
CRP: C-reactive protein
KD: Kawasaki disease
IVIG: intravenous immune globulin
ROB: risk of bias
CHARM: Critical Appraisal and Data Extraction for Systematic Reviews of Prediction Modeling Studies
PRISMA: Preferred Reporting Items for Systematic Reviews and Meta-analyses
TRIPOD: Transparent Reporting of a Multivariable Prediction Model for Individual Prognosis or Diagnosis
AUC: area under the curve of a receiver operating characteristic curve
EPV: event per variable.
