# Supplementary material for: Prediction Model Risk-of-Bias Assessment Tool for coronary artery lesions in Kawasaki disease
Source: Front Cardiovasc Med. 2022 Oct 13;9:1014067. doi: 10.3389/fcvm.2022.1014067 (PMC9606216; doi:10.3389/fcvm.2022.1014067)
Supplement: Supplementary file 1 [file Data_Sheet_1.pdf]

Supplementary material

Prediction Model Risk-of-Bias Assessment Tool for coronary artery lesions in Kawasaki disease

Contents:

Supplementary Table 1 Overview of the primary datasets used in included studies
Supplementary Table 2 Overview of coronary artery lesions prediction models in Kawasaki disease

Supplementary Table 1 Overview of the primary datasets used in included studies

Overview of the primary datasets used in included studies								
Authors	Description of dataset	Study design	Age mean ± SD/ median(IQR)	Sex (% male)	Modelling technique	Total number of CALs	Diagnostic criteria of KD	Diagnostic criteria of CALs
1986-Nakano et al	The Divisions of Pediatric Cardiology and Infectious Diseases, Shizuoka Children's Hospital, Shizuoka, Japan.From May 1977 to October 1985.	Retrospective cohort	Low risk group(year):2 3/12±1 8/12; High risk group(year): 1 5/12±1 1/12	Low risk group:36/62(58.1%); High risk group: 7/16(43.8%)	Discrimination analysis	16/78	Unclear	Low risk group:coronary diameter unded 4mm; High risk group: coronary diameter over 4mm
2007-Kim et al	The Korea University Medical Center in Korea. From March 2001 to February 2005.	Retrospective cohort	Non-CALs was 29.2 months ±20.1; CALs was 35.2 months ±26.9	58.90%	Logistic regression	19/285	AHA2001	Aneurysms were categorised as small when the lumen diameter was 4- 5 mm, moderate when the lumen diameter was 5–8 mm, and large when the lumen

								diameter was > 8 mm.
2013-Ruan et al	The Children's Hospital of Chongqing Medical University in China, From January 2003 to December 2009.	Retrospective cohort	Non-CALs was 33.5months ± 24.2 (27.0); CALs was 31.5months ± 24.2 (26.5)	868/1345(64.5%)	Logistic regression	484/1345	AHA2004	CALs was defined as an internal lumen diameter ≥3 mm in children <5 years of age or ≥4 mm in children >5 years of age
2013-Lega et al	The department of paediatric cardiology of the Lyon Claude Bernard University Medical Centre in France. From January 1988 to June 2007.	Retrospective cohort	Non-CALs was 2.1 years (10 days–14.7 year); CALs was 1.7 years (0.2–14.5 year)	Non-CALs: 70/130(53.8%); CALs: 43/64 (67.2%)	Logistic regression	64/194	AHA2004	CALs were categorised as small (<5 mm internal diameter), medium (5–8 mm) or giant (>8 mm)
2016-Xu et al	The Children's Hospital, Zhejiang University School of Medicine. From August 2009 to December 2012	Retrospective cohort	2.17 years (range 2months to 14.7 years)	276/422(65.4%)	Logistic regression	83/422	Unclear	Coronary artery lesions were defined according to internal lumen diameter of >2.5 mm in children <3 years of age, >3 mm in children of 3–9 years of age;, and >3.5 mm in

								children of 9–14 years of age; internal diameter of a segment measuring ≥1.5 times that of an adjacent segment; and lumen that is clearly irregular.
2016-Kim et al	The Department of Pediatrics, Gachon University Gil Medical Center, Incheon, Korea. From January 2005 to June 2013.	Retrospective cohort	CALs(-) group(month): 33.61±23.65; CALs(+) group(month): 28.61±20.02	CALs(-) group(month): 245/463(52.9%); CALs(+) group(month): 165/240(65.0%)	Logistic regression	240/703	Unclear	CALs was defined as Z-score>2.5 in the RCA, LMCA, LAD, and/or LCX
2017-Berdej-Szczot et al	The Department of Paediatrics, Paediatric Endocrinology, and Diabetes of the Medical University of Silesia, Poland. From May 2003 to March 2016	Retrospective cohort	the age of patients was 1.5–135 months (median age 26 months)	51/73(69.9%)	Logistic regression	13/73	AHA2004	Aneurysms were categorised as small when the lumen diameter was < 5 mm, moderate when the lumen diameter was 5–8 mm, and large when the lumen diameter was > 8 mm.
2017-Liu et al	A tertiary medical	Retrospective	1.4 years (IQR:	99/169(58.6%)	Logistic	31/169	JMH	CALs was difined as

	center in Taiwan. From January 2009 to December 2013	cohort	8.1 months–2.5 years)		regression			maximal Z-score > = +2.5 of any branch of coronary artery
2017-Son et al	a single academic center in USA. From January 1, 2006, through May 1, 2014	Retrospective cohort	3.2 years (IQR1.7, 5.2 years)	169/261(64.8%)	Univariate analysis vs. Logistic regression	93/261	Unclear	CALs was defined as a maximum z score (zMax) ≥2.5 of the left anterior descending or right coronary artery at 4 to 8 weeks of illness
2018 - Kim et al	38 hospitals in Korea. From 2012 to 2014	Retrospective cohort	mean and range: 33 months (1 to 159 months)	2733/5151(53.1%)	Logistic regression	524/5151	AHA2004	CALs was defined as a maximum absolute internal diameter >3 mm in children <5 years of age, or >4 mm in children 5 years and older.
2018-Chantasiriwan et al	The Chiang Mai University Hospital in Thailand. From January 2004 and December 2014	Retrospective cohort	Non-CALs was 18 months (2–79); CALs was 14 months (3–168)	Non-CALs: 104/162(64.2%); CALs: 37/55 (67.3%)	Logistic regression	55/217	AHA2004	A diameter of >2.5 SD for age was defined as CAL.
2018-Hua et al	The Children’s hospital, Zhejiang University School	Retrospective cohort	Median (p25-p75): Non-CALs was 23 months (11–47);	61.50%	Logistic regression	556/2305	JKDRC	CAL were assessed by echocardiography and were defined by

	of Medicine in China. From January 2009 to December 2014		CALs was 19 months (9–33)					either (1) the internal lumen diameter ≥ 2.5 mm in patients aged 0–3, ≥ 3.0 mmin patients aged 3–9, and ≥ 3.5 mmin patients aged 9–14, (2) the internal diameter of a segment ≥ 1.5 times that of an adjacent segment, or (3) the demonstration of clearly irregular lumen
2019-Fernandez-Cooke et al	Total of 84 Spanish hospitals in Spain. From May 2011 to June 2016	Retrospective cohort	49 days to 15.6 years (median: 2.8 years)	63.00%	Logistic regression	144/625	AHA2004	CALs were classified as small (<5-mm internal diameter), medium (5- to 8-mm internal diameter), or giant (>8-mm internal diameter)
2019-Son et al	Development Cohort: The Boston Children's Hospital in USA from January	Retrospective cohort	Development Cohort (Median(Range)): 2.8 years (0.1–15.5); Validation	Development Cohort: 566/903 (62.7%); Validation Cohort: 115/185 (62.2%)	Logistic regression	142/1088	AHA2017	Children were classified as having the outcome of CAL if they had a Z score ≥2.5 in either the LAD

	2006 to May 2014 and the the University of California at San Diego from July 1989 to May 2014. Validation Cohort: a National Heart, Lung, and Blood Institute Pediatric Heart Network clinical trial at 8 North American centers		Cohort: 3.0 years (0.2–12.3)					artery or RCA between 2 and 8 weeks after illness onset.
2020-Turkucar et al	A tertiary center in Turkey. From 1996 to 2018	Retrospective cohort	35 months (19-52)	55/94(58.5%)	Univariable analysis	31/94	JMH	Coronary artery aneurysms were defined as follows: Small aneurysm: localized dilatation showing an inner diameter ≤ 4 mm (in children ≥5 years: the internal diameter of a segment < 1.5 times compared to an adjacent segment).

								Medium aneurysm: aneurysm showing an inner diameter > 4 mm and < 8 mm (in children ≥5 years: the internal diameter of a segment 1.5~ 4 times compared to an adjacent segment). Giant aneurysm: aneurysm showing an internal diameter ≥ 8 mm (in children ≥5 years: the internal diameter of a segment > 4 times compared to an adjacent segment)
2020-Chang et al	The Kaohsiung Chang Gung Memorial Hospital in Taiwan. From 2007 to 2018	Retrospective cohort	Non-CALs was 1.5 years (0.8–2.6); CALs was 1.4 years (0.7–2.4)	Non-CALs: 128/238(53.8%); CALs: 92/127(72.4%)	Logistic regression	127/365	JMH	CALs was defined as: an internal lumen diameter greater than 3.0 mm in children of < 5 years of age or greater than 4.0 mm in children of ≥ 5 years of age or an internal

								diameter at least 1.5 times larger than the diameter of the adjacent segment, or if the morphology of the coronary lumen was obviously irregular, or a z-score ≥ 2.5
2020-Azuma et al	The Minoh City Hospital in Japan. The first study: between March 2002 and 2005, between July 2008 and April 2012. The second study: between July 2014 and December 2018.	Retrospective cohort	First study:30.5 (2.5), Second study: 37.8 (1.8).	First study:64/106 (60.4%), Second study: 121/208 (58.2%).	mean structure equation model (SEM) and neural networks (Nnet)	Unclear	Unclear	CALs was defined as a maximum z score of ≥ 3.0 for the LMT, LAD or RCA.
2021-Huang et al	The MacKay Children's Hospital in Taiwan. From January 1, 2012, through December 31,	Retrospective cohort	Median (p25-p75): Non-CALs was 17.4 months (9.9–26.9); CALs was 16.9 months (8.0–33.2)	Non-CALs: 153/283 (54.1%); CALs: 25/31 (80.6%)	Logistic regression	31/314	AHA2017	CALs was defined as a Z score≥2.5 in the LMCA, LAD, or RCA at 11–60 days after illness onset

	2018							
2021-Iio et al	Data from prospective cohort study performed at 34 hospitals in Japan. From July 1, 2012 to June 30, 2015	Retrospective cohort	Non-CALs was 24 months(14-43); CALs was 12 months (8-23)	Non-CALs: 875/1542 (56.7%); CALs: 53/90 (58.9%)	Logistic regression	90/1757	JKDRC	CAL was defined as a Z score of ≥ 2.5

SD: standard deviation; KD: Kawasaki disease; CALs: coronary artery lesions; IQR: interquartile range; RCA: right coronary artery; LMCA: left main coronary artery; LAD: left anterior descending artery; LCX: left circumflex artery; LMT: left main coronary AHA: American Heart Association; JMH: Japanese Ministry of Health; JKDRC: Japan Kawasaki Disease Research Committee.

Supplementary Table 2 Overview of coronary artery lesions prediction models in Kawasaki disease

Overview of prediction models for Risk Factors of Coronary Artery Aneurysms in Kawasaki Disease							
				Predictive performance on validation			
Study; setting; evaluation method; testing time	Predictors in final model	numbers of candidate predictor	Sample size: total	Type of validation	Sample size: total No of participants for model validation (No with outcome)	Performance*(C index, sensitivity (%), specificity (%), PPV/NPV (%), calibration slope, other (95% CI , if reported))	Overall risk of bias using PROBAST
1986-Nakano et al; Data from Japan general population; diameter; < 3 month	Age, C-reactive protein, platelet count	10	78	Not reported	Not applicable	Sensitivity was 87.5% and specificity was 87.1%	High
2007-Kim et al; Data from Korea general population; diameter; onset-8 weeks	Total days of fever>8d	16	285	Not reported	Not applicable	More than 8 days of total duration of fever (OR=4.1, 95% CI=1.2–14.3)	High
2013-Ruan et al; Date from China general population; diameter; onset-4weeks	Age, Male, Time of IVIG, IVIG dose, platelet count and ESR	28	1345	Not reported	Not applicable	Age (<6 months)(OR=2.0; 95%CI:1.4–2.8), Male(OR=1.6; 95%CI:1.2–2.0), Time of IVIG(OR=3.5; 95%CI:2.1–5.8), IVIG dose(1 g/kg, OR=0.3; 95%CI:0.20–0.42 and 2g/kg, OR=1.8; 95%CI:1.1–3.0), platelet count(OR=1.5; 95%CI:1.2–1.8) and ESR(OR=1.4; 95%CI:1.1–	High

						1.9)	
2013-Lega et al;Date from France general population; diameter; 6-8 weeks	Male, Age, Pericardial effusion, Hemoglobin, IVIG resistance	13	194	Not reported	Not applicable	Age (OR=3.7; 95%CI:1.4–9.9), Sex(OR=3.0; 95%CI:1.1 - 8.0), Pericardial effusion (OR=5.3; 95%CI:1.5–18.0), Hemoglobin(OR=0.6; 95%CI:0.4–0.8) and IVIG resistance(OR=23.6; 95%CI:2.5–226.9)	High
2016-Xu et al; Date from China general population; diameter; 3 months	Red blood cell distribution width (>14.55%), IVIG resistance,Fever duration (>14 days)	25	442	Not reported	Not applicable	Fever duration >14 days (OR = 3.4, 95% CI: 1.3–9.2), intravenous immunoglobulin resistance (OR = 2.3, 95% CI: 1.0–5.3), and red blood cell distribution width >14.55% (OR = 3.5, 95% CI: 2.0–6.1)	High
2016-Kim et al; Data from Korea general population; Z score; 6-8 weeks	Sex, Total fever duration, C-reactive protein, White Blood cells	25	703	Not reported	Not applicable	Male gender (OR=1.7; 95% CI: 1.2-2.3), fever duration of 8 or more days (OR=1.5; 95% CI: 1.1-2.2), CRP≥7 mg/dL (OR=1.7; 95% CI: 1.2-2.3), and WBC count>12×103/μL (OR=1.7; 95% CI: 1.2-2.4)	High
2017-Berdej-Szczot et al; Data from Poland general population; diameter; >one	The day of beginning of treatment, the number of symptoms,	12	73	Not reported	Not applicable	Sensitivity was 85%, specificity was 90%, accuracy was 88.8%	High

month	the maximal platelet						
2017-Liu et al; Data from Taiwan general population; Z score，one month	Initial maximal coronary Z-score of ≥ + 2.5, hypoalbuminemia	12	169	Not reported	Not applicable	Z-score of ≥ + 2.5(OR=5.2,; 95% CI: 1.3–21.3); hypoalbuminemia(OR=1.7; 95% CI: 1.1-20.9).	High
2017-Son et al; Data from North American population; Z score, 4-8 week	baseline zMax ≥2.0	12	261	Not reported	Not applicable	C index 0.77, Hosmer-Lemeshow, P=0.89 Sensitivity 80%,Specificity 74%, Positive Predictive Value 16%, Negative Predictive Value 98%	High
2018-Kim et al; Data from Korea population; diameter, unclear	C-reactive protein	10	5151	Not reported	Not applicable	OR=1.0, 95%CI: 1.0-1.1, AUC: 0.517, Sensitivity: 25.0, Specificity: 81.0, PPV: 0.87, NPV: 0.01	High
2018-Chantasiriwan et al; Data from Thailand population; diameter, 6-8 week	Duration of fever≥8 days and platelet count ≥550 × 109/L	24	217	Not reported	Not applicable	Duration of fever≥8 days(OR 2.8, 95% CI 1.3–6.4); platelet count ≥550 × 109/L(OR 3.0, 95% CI 1.2–6.3)	High
2018-Hua et al; Data from China general population; diameter，2-3 weeks	Male, total fever duration ≥ 8 days, IVIG resistance (IVIGR), albumin (ALB) ≤ 35.9 g/L, eosionphils (EO) ≥ 2.2%, and monocytes (MO) ≥ 5.9%	32	2305	Not reported	Not applicable	Male (OR=1.5 95%CI: 1.2–1.8), total fever duration ≥ 8 days(OR=1.8 95%CI: 1.4–2.2), IVIG resistance (IVIGR)(OR=1.4 95%CI: 1.1–1.9), albumin (ALB) ≤ 35.9 g/L (OR=1.5 95%CI: 1.2–1.9), eosionphils (EO) ≥ 2.2% (OR=1.2 95%CI: 0.9–1.5), and	High

						monocytes (MO) ≥ 5.9% (OR=1.4 95%CI: 1.1–1.7). Sensitivity was 51.4% and specificity was 68.2%, with an AUC of 0.634.	
2019-Fernandez-Cooke et al, Data from Spain population;diameter; at least 6 months	length under 103 cm, hemoglobin < 10.2 mg/dL, platelets > 900,000 cells/mm3, maximum temperature < 39.5˚C, total duration of fever > 10 days and fever before treatment ≥ 8 days	37	625	Not reported	Not applicable	length under 103 cm; OR = 4.9 (CI 95%: 1.3–31.7), hemoglobin < 10.2 mg/dL; OR = 2.2 (CI 95%: 1.2–4.1), platelets > 900,000 cells/mm3; OR = 3.3 (CI 95%: 1.5-7.0), maximum temperature < 39.5˚C; OR = 2.8 (CI 95%: 1.1–9.7), duration of fever > 10 days; OR = 4.8 (CI 95%: 2.5–9.1) and fever before treatment ≥ 8 days OR: 3.9 (CI 95%: 2.1-7.5])	High
2019-Son et al; Data from North American population; Z score, 2 and 8 week	baseline Z score of left anterior descending or right coronary artery ≥2.0, age <6 months, Asian race, and C-reactive protein ≥13 mg/dL	24	903	External validation	185	The model had C index 0.82 (Hosmer-Lemeshow, P=0.816); Validation Cohort revealed C index 0.93 (Hosmer-Lemeshow, P=0.211); The odds of CALs were 16-fold greater in the high(≥3)-versus the low-risk(≤1) groups in the development cohort (OR=16.4; 95% CI, 9.71–27.7), and >40-fold greater in the validation cohort (OR=44.0; 95%	High

						CI, 10.8–180)	
2020-Turkucar et al; Data from Turkey population; diameter; two weeks after IVIG	duration of fever≥9.5 days before IVIG and Plt count after IVIG ≥ 670x103/uL	22	94	Not reported	Not applicable	duration of fever≥9.5 days before IVIG (OR=3.41, sensitivity was 51.6% and specificity was 71.4%), and Plt count after IVIG ≥ 670x103/uL (OR=5.5 sensitivity was 35.7% and specificity was 95.3%) for CALs.	High
2020-Chang et al; Data from Taiwan population; diameter;acute stage	CRP > 103 mg/L, NLR > 3.5, male gender, and IVIG resistance	24	365	Not reported	Not applicable	Sensitivity was 60.8%, and the specificity was 70.6%, with an AUC of 0.696, (OR=3.8, 95% CI 2.4–5.9)	High
2020-Azuma et al; Data from Japan population; Z score; before the treatment, immediately after the treatment and at hospital discharge	age, sex, intravenous immunoglobulin resistance, number of steroid pulse therapy sessions, C-reactive protein level, and urinary β2-microglobulin	11	314	External validation	38	Sensitivity was 73%, specificity was99% and C-index 0.86	High
2021-Huang et al; Data from Taiwan population; Z score; 11–60 days of illness	Male, C-reactive protein, and baseline Zmax	15	314	Not reported	Not applicable	Sensitivity 81% (95%CI: 63%–91%), Specificity 82% (95%CI: 76%–85%), PPV 32% (95%CI: 23%–44%), NPV 97% (95%CI: 95%–99%), False positive rate 18% (95%CI: 14%–23%)	High

2021-Lio et al; Data from Japan population; Z score>2.5; one month	a baseline maximum Z score of >2.5, age of <12 months at fever onset, and nonresponsiveness to IVIG	15	1632	Not reported	Not applicable	Accuracy 82% (95%CI: 77%–86%), Positive likelihood ratio 4.39 (95%CI: 3.25–5.92), Negative likelihood ratio 0.24 (95%CI: 0.12–0.49), AUC of ROC 0.86 (95%CI: 0.78–0.98)"	High

PPV: positive predictive value; NPV: negative predictive value; CI: confidence interval; PROBAST: prediction model risk-of-bias assessment tool; IVIG: intravenous immune globulin; ESR: equivalent series resistance; OR: odds ratio; CRP: C-reactive protein; NLR: neutrophil to lymphocyte ratio; CALs: coronary artery lesions; AUC: area under the curve of a receiver operating characteristic curve; ROC: receiver operating characteristic curve;
